# Measurement of Three-Dimensional Structural Displacement Using a Hybrid Inertial Vision-Based System

**DOI:** 10.3390/s19194083

**Published:** 2019-09-21

**Authors:** Xinxiang Zhang, Yasha Zeinali, Brett A. Story, Dinesh Rajan

**Affiliations:** 1 Department of Electrical and Computer Engineering, Southern Methodist University, Dallas, TX 75205, USA; xinxiang@mail.smu.edu (X.Z.);; 2 Department of Civil and Environmental Engineering, Southern Methodist University, Dallas, TX 75205, USA; yhajizeinali@mail.smu.edu

**Keywords:** three-dimensional, static structural displacement measurement, structural health monitoring, out-of-plane, vision-based, camera calibration, camera movement compensation, motion sensor

## Abstract

Accurate three-dimensional displacement measurements of bridges and other structures have received significant attention in recent years. The main challenges of such measurements include the cost and the need for a scalable array of instrumentation. This paper presents a novel Hybrid Inertial Vision-Based Displacement Measurement (HIVBDM) system that can measure three-dimensional structural displacements by using a monocular charge-coupled device (CCD) camera, a stationary calibration target, and an attached tilt sensor. The HIVBDM system does not require the camera to be stationary during the measurements, while the camera movements, i.e., rotations and translations, during the measurement process are compensated by using a stationary calibration target in the field of view (FOV) of the camera. An attached tilt sensor is further used to refine the camera movement compensation, and better infers the global three-dimensional structural displacements. This HIVBDM system is evaluated on both short-term and long-term synthetic static structural displacements, which are conducted in an indoor simulated experimental environment. In the experiments, at a 9.75 m operating distance between the monitoring camera and the structure that is being monitored, the proposed HIVBDM system achieves an average of 1.440 mm Root Mean Square Error (RMSE) on the in-plane structural translations and an average of 2.904 mm RMSE on the out-of-plane structural translations.

## 1. Introduction

Monitoring the displacements of a structure can provide significant insights into its structural behavior, operating condition, and health [1]. In recent years, accurate measurement of the structural responses under different field conditions has presented a challenging task. This challenging task requires large arrays of instrumentations and incurs high costs in the measurement process. To address this challenging task, several structural health monitoring (SHM) methods focus on monitoring structural acceleration [2,3], but these acceleration-based measurements are typically not accurate when the structural dynamic responses are in the low-frequency ranges. Global positioning systems (GPS) have been investigated by several researchers for measuring static structural displacements. However, these GPS technologies only provide accurate positionings for structures with large displacements, e.g., long-span bridges [4]. Some researchers have used a laser scanning technique [5], but it is not cost-efficient. Meanwhile, some sensor-based techniques have also been applied to monitor structural health and detect structural damage, including radar sensors [6], Fiber Bragg grating (FBG) sensors [7,8], optical fiber sensors [9,10,11], and piezoelectric wafer active sensors [12]. However, those sensor-based techniques usually require direct field installations, which might not be convenient when the monitoring structures, i.e., bridges, have limited access. Therefore, one of the most recent attempts to overcome the limitations of using direct sensor-based techniques is the use of indirect drive-by approaches [13], where the utilized sensors, e.g., lasers, are mounted on passing vehicles to detect the presence and location the bridge damage [14,15]. In such drive-by approaches, bridge scour damages are detected [16,17]. Also, by using these drive-by methods, bridge frequency can be identified [18]. Although these drive-by approaches have shown promising results in the last decade, vehicle-dependent problems arise with this methodology. Due to the increasing development of computer vision in industrial technology [19,20,21], along with visual analysis [22,23,24], indirect vision-based structural displacement measurement systems have rapidly emerged as an alternative for SHM of civil infrastructures. The most representative literature reviews regarding using vision-based technologies are included in [25,26,27], where the reviews cover dynamic response measurements for damage detection. The interactions between vision-based with drive-by approaches were originally provided in a structural identification (St-Id) framework for damage detection and localization [28]. In addition to damage detection [29], the vision-based systems also perform well on structural anomaly detection [30] and traffic monitoring [31]. Compared with the aforementioned SHM systems for measuring structural displacements, the major advantages of these vision-based measurement systems include their cost efficiency, ease of facility setup, and flexibility in extracting displacements of the feature points within certain or multiple region of interests (ROIs) [32] of the structure that is being monitored. Moreover, the vision-based systems can be applied to SHM for modal analysis through monitoring of modal parameters, e.g., modal frequencies [33,34]. In such a context, a recent Motion Magnification (MM) algorithm provided promising results in modal identification of a full-scale historic bridge by using videos taken from a common smartphone device [35].

Specifically, for measuring both dynamic and static structural displacements, these vision-based displacement measurement systems can be broadly classified into target-less and target-based systems. One of the most representative articles regarding noncontact SHM using vison-based systems is [36], in which the performance of both the target-less and target-based systems was analyzed and validated. In the target-less systems, the displacements of distinct features of the monitored structure, such as the corners or the edges, are detected and tracked by computer-vision techniques [37,38,39].

The performance of target-less systems is sensitive to various effects, such as ambient illumination, camera lens distortion, and uncertainties in the displacement directions of the structures. A common limitation of target-less systems is that the ambient illumination should remain unchanged in the measurements; otherwise, motion may be falsely perceived due to the changes in illumination, and be interpreted as structural displacement [40,41]. Therefore, to improve measurement accuracy and to ensure robustness in the field conditions, many industrial SHM applications have been designed based on target-based systems, where multiple calibration targets with distinct features, e.g., checkerboards, are mounted on the surface of the structures to enhance the distinctiveness of the features in the acquired images. In general, using target-based systems can provide more reliable and accurate displacement measurements than using target-less systems, in situations such as when addressing light-induced measurement under extreme field conditions with strong sunlight [42].

Many vision-based structural displacement measurement systems use different characteristics of the imaging system. Most works use monocular camera systems to measure the structural displacements that are parallel to the imaging plane [39,41,43]. These works focus mainly on detecting the in-plane structural translations. To measure the structural displacements perpendicular to the imaging plane, stereo or binocular cameras [44,45,46], depth camera [47,48], and monochrome high-speed cameras [49], have been widely used. However, these depth and high-speed cameras are typically more expensive than monocular cameras, and stereo-camera systems always require accurate image synchronization and registration.

Another common assumption of recent vision-based displacement measurement systems is that the utilized camera is assumed to be stationary during the SHM process. However, in many outdoor SHM processes, it may be difficult to ensure that the camera is stationary during the entire monitoring process. For example, in bridge SHM applications, the cameras are installed at a bridge pier in order to monitor the bridge pivot pier. Due to the translations and rotations of both the bridge pier and the bridge pivot pier, the movements of the monitoring cameras and the bridge pivot pier are subject to both translations and rotations. The camera is displaced, and rotates along with the bridge pier, which will affect the displacement measurements of the bridge pivot pier. In recent years, several camera compensation methods have been developed to compensate the camera movements in SHM and infer the global structural displacements [39,41,43]. However, those methods consider structural displacements to be in-plane translations, i.e., structural displacements parallel to the imaging plane. The out-of-plane translations, i.e., structural displacements that are perpendicular to the imaging plane, are not explicitly included. One of the reasons that these out-of-plane translations are not considered in the SHM process is that the measurement errors from the current vison-based approaches are significant when the structures being monitored are subjected to out-of-plane translations [43]. Although some recent work [50] proposes a vision-based system to measure the out-of-plane translations, camera movement during the SHM has not been well studied.

Additionally, some compensation methods consider camera movements as pure translations, without rotation, during the measurements [39,41]. Errors from such methods may arise if a camera is placed on a platform that has a rotation, such as UAV-based SHM approaches [51,52,53]. Instead of installing the monitoring cameras on the bridge pier, these UAV-based SHM approaches might overcome the limitations of the camera deployment, while presenting another scenario in which translations and rotations need to be compensated.

To accurately measure structural displacements that are subject to both in-plane and out-of-plane translations while considering the rotations and translations of the camera itself in the measurements, this paper presents a target-based HIVBDM system using a monocular CCD camera that is located in the near distance of the monitored pivot pier, such that the structural displacements of the pivot pier can be captured using this CCD camera, and then estimated by using a backbone camera calibration algorithm [54]. The proposed HIVBDM system addresses the challenges described by prior works and develops the methodology in multiple dimensions. A further refinement of the method is developed that couples the camera with a tilt sensor to improve the displacement measurement accuracy of the system, especially in the direction perpendicular to the imaging plan, i.e., out-of-plane translations.

The contributions of the proposed HIVBDM system are: (1) The HIVBDM system is able to measure both the in-plane and out-of-plane structural translations. (2) The HIVBDM system does not require the utilized camera to be stationary during the image acquisition (monitoring) process. The method utilizes multiple targets, at least one of which is placed on a stationary surface within the camera’s FOV, to compensate for the camera movements (both rotations and translations), and accurately infer the global displacements of the structure under study. (3) The robustness of the system is improved, especially with regard to rotations of the camera, by utilizing a tilt sensor that is attached to the camera and provides accurate synchronized rotational information about the camera itself. Even with the attached tilt sensor, the camera translations are still determined by using the stationary calibration target. This additional camera rotational information allows the proposed HIVBDM system to better compensate for the camera’s own movements and infer the global displacements of the structure.

To the best of our knowledge, we are the first to propose a monocular HIVBDM system that accurately measures both the in-plane and out-of-plane structural translations using a moving camera, while considering both the camera’s own rotations and translations. The proposed HIVBDM system incorporates a novel constrained optimization algorithm into the camera calibration process, where the synchronized camera rotations obtained from the attached tilt sensor are added as the optimization constraints. In the meantime, a computational framework for measuring structural displacements aided by a stationary calibration target and an attached tilt sensor is provided. These added constraints regularize the original optimization process of estimating the extrinsic camera parameters, i.e., rotations and translations, and hence improve the accuracy of measuring the global structural displacements.

The remainder of this paper is organized as follows. Section 2 provides an overview of the proposed HIVBDM system and introduces the notations used throughout this paper. Section 3 describes the main procedures and designs of the proposed HIVBDM system. The experimental results are provided in Section 4, and the conclusions are stated in Section 5.

## 2. The Proposed HIVBDM System Overview

**Figure 1:**
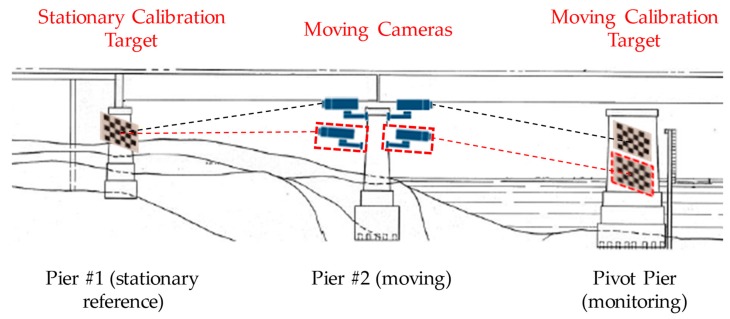
The overview of the proposed HIVBDM system in monitoring a swing bridge pivot pier. A stationary calibration target is mounted to the stationary reference pier, #1. The movements of the cameras and the moving calibration target are subject to the moving pier, #2, and the pivot pier, respectively. We assume that there is no relative movement between the two installed cameras.

In this section, we provide an overview of the proposed HIVBDM System. As shown in Figure 1, the proposed HIVBDM system is motivated by the fact that the pivot pier is the structure that is being monitored, and the reference pier #1 is stationary and will be negligibly displaced and rotated, as it rests upon a solid bedrock foundation; pier #2 and the pivot pier are located in the waterway and are prone to settlement during the bridge’s service life. Installation of cameras on reference pier #1 provides stability for the monitoring camera, but long distances between reference pier #1 and the pivot pier, and a lack of clear line of sight preclude such a solution in application. Shorter distances and a clear line of sight exist between moving pier #2 and both reference pier #1 and the pivot pier. Due to the movements of pier #2, the proposed HIVBDM system should include a scheme that can compensate the movements of pier #2, and hence measure the displacements of the pivot pier. Typically, the proposed HIVBDM system can be developed into a hybrid system where N consecutive bridge piers between the stationary reference pier and the pivot pier are used during the measurement. This designed HIVBDM system is able to address the limitations of the camera lenses at large operating distances and the indirect line of sight between the stationary reference pier and pivot pier in the field. However, the measurement errors between two adjacent bridge piers in the HIVBDM system accumulate with the increasing number of bridge piers that are involved in the measurement.

Before the measurement, since the natural features of the pivot pier are not distinct and might be affected by different effects, i.e., illuminance, shadows, etc., a mounted calibration target (referred as the moving calibration target in Figure 1, is used to enhance the features of the pivot pier. The displacements of the pivot pier are then obtained from this mounted calibration target by assuming that the movements of this calibration target are all subject to the pivot pier. Meanwhile, to capture the camera movements (movements of pier #2) in the SHM process, a second calibration target is mounted to reference pier #1 (referred to as the stationary calibration target in Figure 1) and a second camera that faces reference pier #1 is used (since reference pier #1 is stationary, and the obtained movements are all from the moving camera). By combining the corresponding structural displacements and camera movements from these two monitoring cameras (we assume that there is no relative movement between these two cameras), the displacements of the pivot pier can eventually be measured. During the measurement, the structural rotation responses are usually minimal compared with the structural translation responses [39,43]; hence, the structural rotation responses of the pivot pier are usually not taken into consideration in the field. However, those minimal structural rotation responses of the moving pier #2 (used to install the two monitoring cameras) are usually considered in the measurement since any minimal “uncorrected” rotation of the moving pier #2 might deteriorate the measurement accuracy, especially in the large operation distance between the moving pier #2 and the pivot pier. Therefore, in the proposed HIVBDM system, the structural translations on both the moving pier #2 and the pivot pier are considered in the measurement, but only the structural rotations on the moving pier #2 are included. Since we assume that there is no relative movement between the two installed monitoring cameras, the effective model of the proposed HIVBDM system consists of one moving camera, one stationary calibration target, and one moving calibration target. To substitute the usage of pier #1 as a stationary reference in the field applications, the stationary and moving calibration targets are required to be located within the same FOV of the moving camera in each of the monitoring images.

Based on the above motivations, the proposed HIVBDM system is then designed such that the three-dimensional displacements, e.g., translations, of the pivot pier are measured, while considering the camera’s own movements, e.g., three-dimensional translations and rotations from the moving pier #2. The input of the proposed HIVBDM system is the image sequence that captures features of the structure being monitored at the pixel level, and the output of the system is the measured three-dimensional structural displacements in the world unit. The backbone of the proposed HIVBDM system is the target-based camera calibration algorithm [54].

This HIVBDM system is then evaluated on the basis of experiments simulating static bridge displacements that are performed in an indoor experimental environment. Generally, this proposed HIVBDM can be extended to measure dynamic responses of the structures provided a suitable camera with a high acquisition frame rate.

Considering the practical field operating distances between the camera pier and pivot pier, and the limited laboratory space, the operating distance between the camera and structure (calibration target) is set at 9.75 m throughout the experiments. The experimental results indicate that by using a stationary calibration target to compensate the camera movements, RMSE of approximately 8 mm and 12 mm are achieved on the measured in-plane and out-of-plane translations, respectively. By using an attached tilt sensor, the RMSE is reduced to less than 2 mm on in-plane translations and around 3 mm on out-of-plane translations. The frequently used notations in this paper are provided in Table 1 and the details of the proposed HIVBDM system design are discussed in Section 3.

## 3. Procedures and Designs of the Proposed HIVBDM System

In this section, we provide the details of the proposed HIVBDM system. There are three main procedures in this proposed HIVBDM system: (1) The relative displacement measurements between the camera and the structure being monitored using a stationary camera are proposed in Section 3.1. (2) The relative displacement measurements between the camera and structure being monitored using a moving camera are presented in Section 3.2. While the camera is moving, the measurements utilize a stationary calibration target to capture the camera movements, i.e., both translations and rotations, and infer the global structural displacements. (3) In addition, the utilization of an attached tilt sensor is provided. Since the camera rotations captured by the stationary calibration target have reduced accuracy with the increasing operating distances, an attached tilt sensor is supplemented in order to refine the camera rotations and improve the measurements. Instead of only using the stationary calibration target for capturing both the camera translations and rotations, the attached tilt sensor is only used to measure the camera rotations, where a stationary calibration target is still required for capturing the camera translations.

### 3.1. Relative Displacement Measurements between the Camera and Structure Using a Stationary Camera

**Figure 2:**
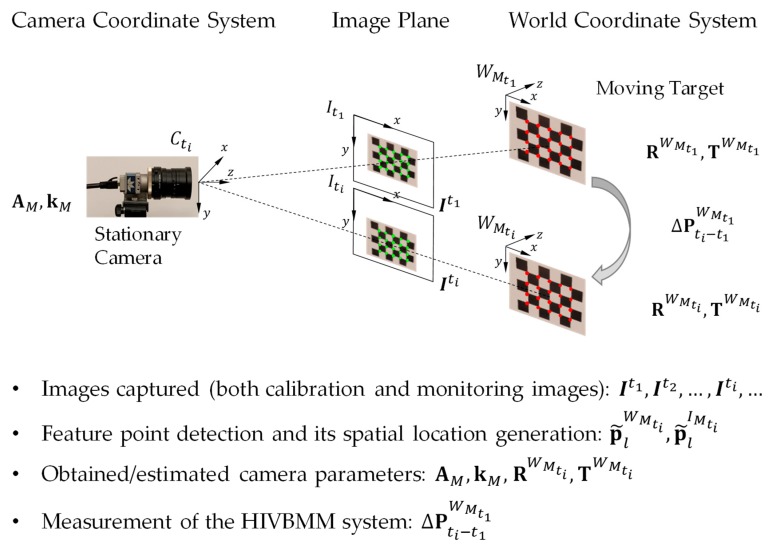
Illustration of structural displacement measurements using a stationary camera. The moving calibration target is assumed to have the same movements with the structure that is being monitored. The calibration images (need to cover the whole camera FOV) are taken before the monitoring images. For better visualization, only the monitoring images $I^{t_{1}}$, $I^{t_{i}}$ are shown.

In this section, since the camera is stationary, the relative displacement between the camera and the monitored structure represent the structural displacement. The measurement system using a stationary camera is shown in Figure 2, where the system includes a stationary camera and a calibration target mounted to the structure that is being monitored. We assume that there is no relative movement between the mounted calibration target and the monitored structure. For simplicity, only the calibration target is shown in Figure 2. Two reference coordinate systems and one plane at time $t_{i}$ are included in the HIVBDM system: (1) The world coordinate system of the moving structure (calibration target) at time $t_{i}$, i.e., $W_{M_{t_{i}}}$, (2) the camera coordinate system at time $t_{i}$, i.e., $C_{t_{i}}$, and (3) the image plane at $t_{i}$, i.e., $I_{t_{i}}$.

The input of the HIVBDM system using a stationary camera is the image sequence I with the calibration target on the monitored structure at each frame. The output of the HIVBDM system is the measured three-dimensional structural translations of the monitored structure in the world unit. Please note that the dimension of the calibration target in the world unit is known, and the feature points are distinct in the calibration target, i.e., checkerboard. As shown in Figure 2, the pixel-wise locations of the feature points on the moving calibration target, i.e., green points on the image plane, are detected at the input image $I^{t_{i}}$. The $l^{th}$ detected feature points on the moving calibration target of the input image $I^{t_{i}}$ is denoted as ${\tilde{p}}_{l}^{I_{M_{t_{i}}}}={\left[{\tilde{x}}_{l}^{I_{M_{t_{i}}}},\;{\tilde{y}}_{l}^{I_{M_{t_{i}}}}\right]}^{T}$, where $l\in \left\{1,2,\ldots ,L_{t_{i}}\right\}$ and $L_{t_{i}}$ is the number of detected feature points on the moving calibration target of input image $I^{t_{i}}$. The spatial locations of these detected feature points on the moving calibration target, i.e., red points in the world coordinate system, are generated for the input image $I^{t_{i}}$ based on the prior calibration target dimensions. The origin of the moving calibration target in the world coordinate system is assumed to be ${\left[0,\;0,\;0\right]}^{T}$, and the spacings between the checkerboard corners are known. As a result, the generated spatial location of the $l^{th}$ detected feature point on the moving calibration target of the input image $I^{t_{i}}$ is denoted as ${\tilde{p}}_{l}^{W_{M_{t_{i}}}}={\left[{\tilde{x}}_{l}^{W_{M_{t_{i}}}},\;{\tilde{y}}_{l}^{W_{M_{t_{i}}}},\;{\tilde{z}}_{l}^{W_{M_{t_{i}}}}\right]}^{T}$, where $l\in \left\{1,2,\ldots ,L_{t_{i}}\right\}$.

Based on the pinhole camera model with the radial lens distortion, the relationship between the 3D spatial location ${\tilde{p}}_{l}^{I_{M_{t_{i}}}}$ and the 2D pixel location ${\tilde{p}}_{l}^{W_{M_{t_{i}}}}$ is given by:

(1) $$\left[\begin{array}{c} {\tilde{x}}_{l}^{I_{M_{t_{i}}}}\\ {\tilde{y}}_{l}^{I_{M_{t_{i}}}}\\ 1 \end{array}\right]=F_{k_{M}}\left(A_{M}\cdot \left[R^{W_{M_{t_{i}}}}|T^{W_{M_{t_{i}}}}\right]\cdot \left[\begin{array}{c} {\tilde{x}}_{l}^{W_{M_{t_{i}}}}\\ {\tilde{y}}_{l}^{W_{M_{t_{i}}}}\\ {\tilde{z}}_{l}^{W_{M_{t_{i}}}}\\ 1 \end{array}\right]\right)$$

where $A_{M}$ is the intrinsic camera parameter, $R^{W_{M_{t_{i}}}}$ and $T^{W_{M_{t_{i}}}}$ are the extrinsic camera parameters, F(·) is the radial lens distortion function, and $k_{M}$ is the parameter of this radial lens distortion. The dimensions of those parameters in Equation (1) are given in Table 1.

Given the $L_{t_{i}}$ detected feature points on the moving calibration target of the input image $I^{t_{i}}$ (green points on the image plane of Figure 2, and their generated spatial locations (red points in the world coordinate system of Figure 2, the unknown camera parameters, i.e., $A_{M},k_{M},\;R^{W_{M_{t_{i}}}},T^{W_{M_{t_{i}}}}$ in Equation (1) are obtained from the camera calibration algorithm by minimizing the reprojection error $\left\|\varepsilon_{R}\right\|$ (in the least squares sense) through a non-linear optimization process. The reprojection error $\left\|\varepsilon_{R}\right\|$ over all the feature points of the input image sequence is defined as:

(2) $$\left\|\varepsilon_{R}\|=\sum_{t_{i}=1}^{M+N}\sum_{l=1}^{L_{t_{i}}}\|{\tilde{p}}_{l}^{I_{M_{t_{i}}}}-℘\left(A_{M},k_{M},\;R^{W_{M_{t_{i}}}},T^{W_{M_{t_{i}}}},{\tilde{p}}_{l}^{W_{M_{t_{i}}}}\right)\right\|$$

The ℘(·) is a projection function that maps the 3D spatial location ${\tilde{p}}_{l}^{W_{M_{t_{i}}}}$ to the 2D pixel location ${\tilde{p}}_{l}^{I_{M_{t_{i}}}}$ by using the intrinsic camera parameter $A_{M}$, the extrinsic camera parameters $R^{W_{M_{t_{i}}}}$, $T^{W_{M_{t_{i}}}}$, and the radial lens distortion $k_{M}$. The overall number of input images equals (M+N), where the M calibration images provide sufficient geometric information required for estimating the unknown intrinsic camera parameters, and the N monitoring images capture the structural displacements in the SHM process. Please note that to accurately estimate the unknown intrinsic camera parameters, the M calibration images usually need to cover the entire camera FOV with different orientations.

Since the structure that is being monitored is only subject to translations, and the camera is stationary throughout the monitoring process, the constrained optimization problem is then defined as follows:

(3) $$\underset{A_{M},k_{M},R^{W_{M_{t_{i}}}},T^{W_{M_{t_{i}}}}}{\min}\|\varepsilon_{R}\|\;s.t.\;R^{W_{M_{t_{M+i}}}}=R^{W_{M_{t_{M+1}}}},\;\forall i\in \left\{1,\ldots ,N\right\}$$

The constrained optimization problem is iteratively solved by the Levenberg-Marquardt Algorithm [55], where the initial estimates of the parameters are given in [56]. The optimization process leverages the overall $L_{t_{i}}$ detected feature points ${\tilde{p}}_{l}^{I_{M_{t_{i}}}}$ and their generated spatial locations ${\tilde{p}}_{l}^{W_{M_{t_{i}}}}$ on the moving calibration target from all the (M+N) input images.

Therefore, based on those solved camera parameters, i.e., $A_{M},k_{M},\;R^{W_{M_{t_{i}}}},T^{W_{M_{t_{i}}}}$, from the moving calibration target in Equation (3), the HIVBDM system using a stationary camera then measures the structural displacements $\Delta P_{t_{i}-t_{1}}^{W_{M_{t_{1}}}}$ from $t_{1}$ to $t_{i}$ in the world unit. The measurement process that leverages those obtained extrinsic camera parameters (from the N monitoring images) is provided in Equations (4)–(8).

Since the entire monitored structure is assumed to have the same displacement, a point P on the moving calibration target is selected as the monitored point to represent the overall structural displacements in the measurements. Based on the pinhole camera model in camera calibration and the monitored point P, the relationship between the point locations $P_{t_{i}}^{C_{t_{i}}}$ and $P_{t_{i}}^{W_{M_{t_{i}}}}$ at time $t_{i}$ is given by:

(4) $$P_{t_{i}}^{C_{t_{i}}}=R^{W_{M_{t_{i}}}}P_{t_{i}}^{W_{M_{t_{i}}}}+T^{W_{M_{t_{i}}}}$$

where the $R^{W_{M_{t_{i}}}}$ and $T^{W_{M_{t_{i}}}}$ are the obtained extrinsic camera parameters from the camera calibration. Following Equation (4), the point location $P_{t_{i}}^{W_{M_{t_{1}}}}$ at time $t_{i}$ in $W_{M_{t_{1}}}$ is calculated as:

(5) $$P_{t_{i}}^{W_{M_{t_{1}}}}=R^{W_{M_{t_{1}}}}{}^{-1}\left(P_{t_{i}}^{C_{t_{1}}}-T^{W_{M_{t_{1}}}}\right)$$

Since the camera is stationary, the $P_{t_{i}}^{C_{t_{i}}}\equiv P_{t_{i}}^{C_{t_{1}}}$ is achieved at any time $t_{i}$. Following this stationary camera prior and then substituting $P_{t_{i}}^{C_{t_{1}}}$ in Equation (5) using the right side of Equation (4), the location $P_{t_{i}}^{W_{M_{t_{1}}}}$ at time $t_{i}$ in $W_{M_{t_{1}}}$ is calculated as:

(6) $$\begin{array}{l} P_{t_{i}}^{W_{M_{t_{1}}}}=R^{W_{M_{t_{1}}}}{}^{-1}\left(P_{t_{i}}^{C_{t_{i}}}-T^{W_{M_{t_{1}}}}\right)\\ \;=R^{W_{M_{t_{1}}}}{}^{-1}\left(R^{W_{M_{t_{i}}}}P_{t_{i}}^{W_{M_{t_{i}}}}+T^{W_{M_{t_{i}}}}-T^{W_{M_{t_{1}}}}\right)\\ \;=R^{W_{M_{t_{1}}}}{}^{-1}R^{W_{M_{t_{i}}}}P_{t_{i}}^{W_{M_{t_{i}}}}+R^{W_{M_{t_{1}}}}{}^{-1}\left(T^{W_{M_{t_{i}}}}-T^{W_{M_{t_{1}}}}\right) \end{array}$$

Since the structure that is being monitored is subject to only translations, and the camera is stationary throughout the SHM process, the $R^{W_{M_{t_{1}}}}{}^{-1}R^{W_{M_{t_{i}}}}\equiv I$ is achieved at each time $t_{i}$. Hence, the different selections of the monitored point P are not critical in this study. For simplicity, the origin of the moving calibration target in $W_{M_{t_{i}}}$ is selected as the monitored point P, i.e., $P_{t_{i}}^{W_{M_{t_{i}}}}\equiv {\left[0,\;0,\;0\right]}^{T}$, and the location $P_{t_{i}}^{W_{M_{t_{1}}}}$ in Equation (6) is simplified as:

(7) $$P_{t_{i}}^{W_{M_{t_{1}}}}=R^{W_{M_{t_{1}}}}{}^{-1}\left(T^{W_{M_{t_{i}}}}-T^{W_{M_{t_{1}}}}\right)$$

Hence, the structural displacements between $P_{t_{i}}^{W_{M_{t_{1}}}}$ and $P_{t_{1}}^{W_{M_{t_{1}}}}$ using a stationary camera are calculated as:

(8) $$\Delta P_{t_{i}-t_{1}}^{W_{M_{t_{1}}}}=P_{t_{i}}^{W_{M_{t_{1}}}}-P_{t_{1}}^{W_{M_{t_{1}}}}=R^{W_{M_{t_{1}}}}{}^{-1}\left(T^{W_{M_{t_{i}}}}-T^{W_{M_{t_{1}}}}\right)$$

The $\Delta P_{t_{i}-t_{1}}^{W_{M_{t_{1}}}}$ in Equation (8) is the measurement output of the HIVBDM system using a stationary camera. In addition, when the monitored structure in $W_{M_{t_{1}}}$ is parallel to the imaging plane, i.e., $R^{W_{M_{t_{1}}}}=I$, the measured structural displacements in Equation (8) are simplified to $T^{W_{M_{t_{i}}}}-T^{W_{M_{t_{1}}}}$, where only the translation difference is considered.

### 3.2. Relative Displacement Measurements between the Camera and Structure Using a Moving Camera

Although the camera can be kept stationary in many structural monitoring processes, finding a stationary platform on which to place the camera throughout a long-term monitoring process may not be convenient. Therefore, if both the camera and monitored structure are moving, the relative displacement measurements between the camera and monitored structure described in Section 3.1 may not yield the valid measurement results.

**Figure 3:**
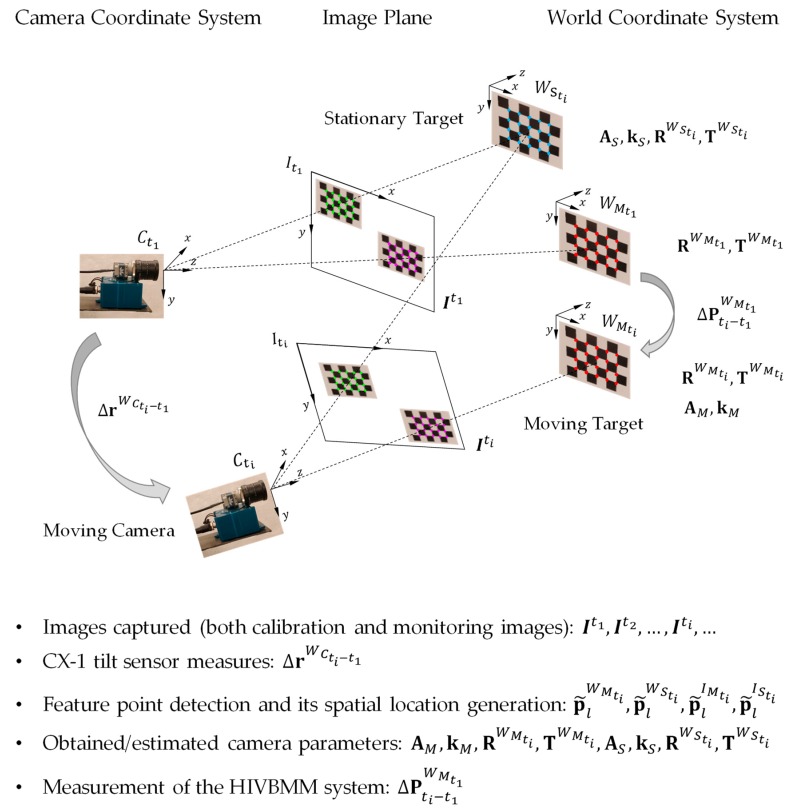
Illustration of structural displacement measurement using a moving camera. The stationary calibration target is assumed to have the same movements with the stationary structure, and the moving calibration target is assumed to have the same movements with the structure that is being monitored. Both the stationary and moving calibration targets are required to place within the same FOV of the camera. The calibration images (need to cover the whole camera FOV) are taken before the monitoring images. For better visualization, only the monitoring images $I^{t_{1}}$, $I^{t_{i}}$ are shown.

In this section, we present a relative displacement measurement method that is able to distinguish the camera movements from the structural displacements by leveraging a novel camera movement compensation method and hence infers the global structural displacements under study. In the camera movement compensation, a calibration target mounted to an additional stationary structure within the same camera FOV is firstly used to capture the camera movements. However, the camera movements captured by the stationary calibration target may not be accurate enough in the applications with increasing operating distances due to the sensitive camera rotation information. Therefore, an attached tilt sensor is utilized to supplement the stationary calibration target in the camera movement compensation process and improves the relative displacement measurement accuracies. The details of the camera movement compensation using a stationary calibration target are presented in Section 3.2.1, and the details of the camera movement compensation using a stationary calibration target with a supplemental tilt sensor attached are then presented in Section 3.2.2. As shown in Figure 3, the measurement system using a moving camera includes a moving monitoring camera (with an attached tilt sensor), a calibration target mounted to a stationary structure (stationary target), and a calibration target mounted to the structure that is being monitored (moving target). These stationary and moving calibration targets are both localized within the same FOV of the camera during the measurements. Similarly, we assume that there is no relative movement between the calibration targets and the mounted structural surface, and only the calibration targets are shown in Figure 3.

Similar to the HIVBDM system geometries described in Figure 2, three reference coordinate systems and one plane at time $t_{i}$ are included in this HIVBDM system: (1) The world coordinate system of the moving structure at time $t_{i}$, i.e., $W_{M_{t_{i}}}$, (2) the world coordinate system of the stationary structure at time $t_{i}$, i.e., $W_{S_{t_{i}}}$, (3) the camera coordinate system at time $t_{i}$, i.e., $C_{t_{i}}$, and (4) the image plane at $t_{i}$, i.e., $I_{t_{i}}$. The inputs of the HIVBDM system using a moving camera are the image sequence I with the calibration targets on both the stationary and the monitored structures at each frame, and the camera rotation information from the attached tilt sensor with each frame (only used in Section 3.2.2). The outputs of the HIVBDM system are the measured three-dimensional structural translations in the world unit.

#### 3.2.1. Camera Movement Compensation Using a Stationary Calibration Target

Unlike the measurement setups shown in Figure 2, an extra calibration target mounted on a stationary structure is used in this series of measurements. As shown in Figure 3, the pixel-wise locations of the feature points on both the stationary calibration target, i.e., green points on the image plane, and on the moving calibration target, i.e., purple points on the image plane, are detected at the input image $I^{t_{i}}$. Specifically, in the input image $I^{t_{i}}$, the $l^{th}$ detected feature point on the stationary calibration target is denoted as ${\tilde{p}}_{l}^{I_{S_{t_{i}}}}={\left[{\tilde{x}}_{l}^{I_{S_{t_{i}}}},\;{\tilde{y}}_{l}^{I_{S_{t_{i}}}}\right]}^{T}$, and that on the moving calibration target is denoted as ${\tilde{p}}_{l}^{I_{M_{t_{i}}}}={\left[{\tilde{x}}_{l}^{I_{M_{t_{i}}}},\;{\tilde{y}}_{l}^{I_{M_{t_{i}}}}\right]}^{T}$, where $l\in \left\{1,2,\ldots ,L_{t_{i}}\right\}$ and $L_{t_{i}}$ is the number of detected feature points on both the stationary and the moving calibration targets. Meanwhile, the spatial locations of these detected feature points on the stationary calibration target, i.e., blue points in the world coordinate system, and those on the moving calibration target, i.e., red points in the world coordinate system, are generated for the input image $I^{t_{i}}$ based on the prior calibration target dimensions. The generated spatial location of the $l^{th}$ detected feature points on the stationary calibration target is denoted as ${\tilde{p}}_{l}^{W_{S_{t_{i}}}}={\left[{\tilde{x}}_{l}^{W_{S_{t_{i}}}},\;{\tilde{y}}_{l}^{W_{S_{t_{i}}}},\;{\tilde{z}}_{l}^{W_{S_{t_{i}}}}\right]}^{T}$, and that on the moving calibration target is denoted as ${\tilde{p}}_{l}^{W_{M_{t_{i}}}}={\left[{\tilde{x}}_{l}^{W_{M_{t_{i}}}},\;{\tilde{y}}_{l}^{W_{M_{t_{i}}}},\;{\tilde{z}}_{l}^{W_{M_{t_{i}}}}\right]}^{T}$, where $l\in \left\{1,2,\ldots ,L_{t_{i}}\right\}$.

Similar to those described in Section 3.1, the relationship between the 3D spatial location ${\tilde{p}}_{l}^{W_{M_{t_{i}}}}$ and the 2D pixel location ${\tilde{p}}_{l}^{I_{M_{t_{i}}}}$ is shown as Equation (1). Given the $L_{t_{i}}$ detected feature points on the moving calibration target of the input image $I^{t_{i}}$ (purple points on the image plane of Figure 3, and their corresponding generated spatial locations (red points in the world coordinate system of Figure 3, the unknown camera parameters, i.e., $A_{M},k_{M},\;R^{W_{M_{t_{i}}}},T^{W_{M_{t_{i}}}}$ in Equation (1), are obtained by minimizing the reprojection error $\left\|\varepsilon_{R}\right\|$ defined in Equation (2). In this study, the estimation of these unknown camera parameters using the moving calibration target is considered to be an optimization problem. Since the camera movements are unknown, the optimization problem is then defined as follows:

(9) $$\underset{A_{M},k_{M},R^{W_{M_{t_{i}}}},T^{W_{M_{t_{i}}}}}{\min}\;\|\varepsilon_{R}\|$$

where the extrinsic camera parameters are subject to rotations (from camera) and translations (from both camera and the moving structure) at any time $t_{i}$. Unlike using the solved camera parameters from the stationary camera in Equation (3), the HIVBDM system using a moving camera is not able to measure the structural displacements $\Delta P_{t_{i}-t_{1}}^{W_{M_{t_{1}}}}$ from $t_{1}$ to $t_{i}$ in the world unit by using those solved camera parameters in Equation (9).

Therefore, to isolate the structural displacements from the camera movements, a stationary structure within the same camera FOV of the structure that is being monitored is used to capture the camera movements on which the relative movements between the camera and the stationary structure (stationary calibration target) are considered as pure camera movements.

Similar to Equation (1), the relationship between the 3D spatial location ${\tilde{p}}_{l}^{W_{S_{t_{i}}}}$ and the 2D pixel location ${\tilde{p}}_{l}^{I_{S_{t_{i}}}}$ is given by:

(10) $$\left[\begin{array}{c} {\tilde{x}}_{l}^{I_{S_{t_{i}}}}\\ {\tilde{y}}_{l}^{I_{S_{t_{i}}}}\\ 1 \end{array}\right]=F_{k_{s}}\left(A_{S}\cdot \left[R^{W_{S_{t_{i}}}}|T^{W_{S_{t_{i}}}}\right]\cdot \left[\begin{array}{c} {\tilde{x}}_{l}^{W_{S_{t_{i}}}}\\ {\tilde{y}}_{l}^{W_{S_{t_{i}}}}\\ {\tilde{z}}_{l}^{W_{S_{t_{i}}}}\\ 1 \end{array}\right]\right)$$

Given the $L_{t_{i}}$ detected feature points on the stationary calibration target of the input image $I^{t_{i}}$ (green points on the image plane of Figure 3, and their generated spatial locations (blue points in the world coordinate system of Figure 3, the unknown camera parameters, i.e., $A_{S},k_{S},\;R^{W_{S_{t_{i}}}},T^{W_{S_{t_{i}}}}$ in Equation (10), are obtained by minimizing the reprojection error $\left\|\varepsilon_{R}\right\|$ (in the least squares sense) through an optimization process, where the $\left\|\varepsilon_{R}\right\|$ is defined as:

(11) $$\left\|\varepsilon_{R}\|=\sum_{t_{i}=1}^{M+N}\sum_{l=1}^{L_{t_{i}}}\|{\tilde{p}}_{l}^{I_{S_{t_{i}}}}-℘\left(A_{S},k_{S},\;R^{W_{S_{t_{i}}}},T^{W_{S_{t_{i}}}},{\tilde{p}}_{l}^{W_{S_{t_{i}}}}\right)\right\|$$

Similarly, ℘(·) is a projection function which maps the 3D spatial location ${\tilde{p}}_{l}^{W_{S_{t_{i}}}}$ to the 2D pixel location ${\tilde{p}}_{l}^{I_{S_{t_{i}}}}$. The M calibration images provide geometric information required for estimating the unknown intrinsic camera parameters, and the N monitoring images capture the structural displacements in the SHM process. In this study, estimating those unknown camera parameters (camera movements) using the stationary calibration target is considered to be an optimization problem. Similar to Equation (9), since the camera movements are unknown, the optimization problem is then defined as follows:

(12) $$\underset{A_{S},k_{S},R^{W_{S_{t_{i}}}},T^{W_{S_{t_{i}}}}}{\min}\;\|\varepsilon_{R}\|$$

where the extrinsic camera parameters are subject to camera rotations and translations at any time $t_{i}$. The solved camera parameters from the stationary calibration target in Equation (12) represent the camera movements.

Therefore, based on the solved camera parameters from the moving and stationary calibration target in Equation (9) and Equation (12), respectively, the HIVBDM system using a moving camera then measures the structural displacements $\Delta P_{t_{i}-t_{1}}^{W_{M_{t_{1}}}}$ from $t_{1}$ to $t_{i}$ in the world unit. The measurement process that leverages those obtained extrinsic camera parameters (both from the N monitoring images) is provided in Equations (13)–(18).

Following Equation (4), considering that the monitored point P is on a moving calibration target, the relationship between the point locations in $W_{S_{t_{i}}}$ and in $W_{M_{t_{i}}}$ at time $t_{i}$ can be shown as:

(13) $$R^{W_{S_{t_{i}}}}P_{t_{i}}^{W_{S_{t_{i}}}}+T^{W_{S_{t_{i}}}}=R^{W_{M_{t_{i}}}}P_{t_{i}}^{W_{M_{t_{i}}}}+T^{W_{M_{t_{i}}}}=P_{t_{i}}^{C_{t_{i}}}$$

where the location of the point P at time $t_{i}$ in $W_{S_{t_{i}}}$ is calculated as:

(14) $$P_{t_{i}}^{W_{S_{t_{i}}}}=R^{W_{S_{t_{i}}}}{}^{-1}\left(R^{W_{M_{t_{i}}}}P_{t_{i}}^{W_{M_{t_{i}}}}+T^{W_{M_{t_{i}}}}-T^{W_{S_{t_{i}}}}\right)$$

Since the world coordinate system of the stationary calibration target at $t_{i}$ remains the same as that at the initial time $t_{1}$, the $P_{t_{i}}^{W_{S_{t_{i}}}}\equiv P_{t_{i}}^{W_{S_{t_{1}}}}$ is achieved at any time $t_{i}$. Following Equation (13), the location of the point P at time $t_{i}$ in $W_{M_{t_{1}}}$ is calculated as:

(15) $$P_{t_{i}}^{W_{M_{t_{1}}}}=R^{W_{M_{t_{1}}}}{}^{-1}\left(R^{W_{S_{t_{1}}}}P_{t_{i}}^{W_{S_{t_{1}}}}+T^{W_{S_{t_{1}}}}-T^{W_{M_{t_{1}}}}\right)$$

Following the stationary calibration target prior, $P_{t_{i}}^{W_{S_{t_{1}}}}\equiv P_{t_{i}}^{W_{S_{t_{i}}}}$, and substituting $P_{t_{i}}^{W_{S_{t_{1}}}}$ using Equation (14), the location $P_{t_{i}}^{W_{M_{t_{1}}}}$ at time $t_{i}$ in $W_{M_{t_{1}}}$ is calculated as:

(16) $$P_{t_{i}}^{W_{M_{t_{1}}}}=R^{W_{M_{t_{1}}}}{}^{-1}\left(R^{W_{S_{t_{1}}}}R^{W_{S_{t_{i}}}}{}^{-1}\left(R^{W_{M_{t_{i}}}}P_{t_{i}}^{W_{M_{t_{i}}}}+T^{W_{M_{t_{i}}}}-T^{W_{S_{t_{i}}}}\right)+T^{W_{S_{t_{1}}}}-T^{W_{M_{t_{1}}}}\right)$$

Since the orientations of the calibration targets regarding the monitoring camera are similar, and only the camera rotations are considered throughout the entire structural monitoring process, the $R^{W_{M_{t_{1}}}}{}^{-1}R^{W_{S_{t_{1}}}}R^{W_{S_{t_{i}}}}{}^{-1}R^{W_{M_{t_{i}}}}\approx I$ is achieved at each time $t_{i}$. Hence, the different selections of the monitored point P are not critical in this study. For simplicity, the origin of the moving calibration target in $W_{M_{t_{i}}}$ is selected as the monitored point P, i.e., $P_{t_{i}}^{W_{M_{t_{i}}}}\equiv {\left[0,\;0,\;0\right]}^{T}$, and the location $P_{t_{i}}^{W_{M_{t_{1}}}}$ in Equation (16) is simplified as:

(17) $$P_{t_{i}}^{W_{M_{t_{1}}}}=R^{W_{M_{t_{1}}}}{}^{-1}\left(R^{W_{S_{t_{1}}}}R^{W_{S_{t_{i}}}}{}^{-1}\left(T^{W_{M_{t_{i}}}}-T^{W_{S_{t_{i}}}}\right)+T^{W_{S_{t_{1}}}}-T^{W_{M_{t_{1}}}}\right)$$

Hence, the structural displacements between $P_{t_{i}}^{W_{M_{t_{1}}}}$ and $P_{t_{1}}^{W_{M_{t_{1}}}}$ using a moving camera are calculated as:

(18) $$\begin{array}{ll} \Delta P_{t_{i}-t_{1}}^{W_{M_{t_{1}}}} & =P_{t_{i}}^{W_{M_{t_{1}}}}-P_{t_{1}}^{W_{M_{t_{1}}}}\\ & =R^{W_{M_{t_{1}}}}{}^{-1}\left(R^{W_{S_{t_{1}}}}R^{W_{S_{t_{i}}}}{}^{-1}\left(T^{W_{M_{t_{i}}}}-T^{W_{S_{t_{i}}}}\right)+T^{W_{S_{t_{1}}}}-T^{W_{M_{t_{1}}}}\right) \end{array}$$

The $\Delta P_{t_{i}-t_{1}}^{W_{M_{t_{1}}}}$ in Equation (18) is the measurement output of the HIVBDM system using a moving camera and a stationary calibration target as camera movement compensation.

#### 3.2.2. Camera Movement Compensation Using a Stationary Calibration Target with an Attached Tilt Sensor

Although the camera movement compensation using a stationary calibration target is able to measure the structural displacements while the camera is moving, the captured camera rotation information using only the stationary calibration target may lead to a reduction in accuracy with increasing operating distances. Camera movement compensation using an attached tilt sensor is therefore leveraged to supplement the stationary calibration target in better capturing the camera movements and infers the global structural displacements. As shown in Figure 3, instead of using a stationary calibration target to capture the camera rotations, the camera rotations are directly obtained by using an attached tilt sensor (the blue CX-1 tilt sensor [57] underneath the camera).

In this section, the measurement process is similar to that described in Section 3.2.1. However, unlike the optimization process in Equation (9) and Equation (12) on both the moving and stationary calibration targets, the obtained camera rotations from the attached tilt sensor are added into the optimization process as the constraints.

Similarly, given the $L_{t_{i}}$ detected feature points on the moving calibration target of the input image $I^{t_{i}}$ (purple points on the image plane of Figure 3), and their corresponding generated spatial locations (red points in the world coordinate system of Figure 3), the unknown camera parameters, i.e., $A_{M},k_{M},\;R^{W_{M_{t_{i}}}},T^{W_{M_{t_{i}}}}$ in Equation (1), are obtained by minimizing the reprojection error $\left\|\varepsilon_{R}\right\|$ defined in Equation (2). In this study, the estimation of these unknown camera parameters using the moving calibration target is considered to be a constrained optimization problem, where the camera rotations are known from the attached tilt sensor, and the structure is subject to only translations. Therefore, the constrained optimization problem is defined as follows:

(19) $$\underset{A_{M},k_{M},R^{W_{M_{t_{i}}}},T^{W_{M_{t_{i}}}}}{\min}\|\varepsilon_{R}\|\;s.t.\;R^{W_{M_{t_{M+i}}}}=R^{W_{M_{t_{M+1}}}}⊕\left(\Delta R^{W_{C_{t_{M+i}-t_{M+1}}}}\right),\;\forall i\in \left\{1,\ldots ,N\right\}$$

where the difference of the rotation matrices of the camera from the moving structure between the time $t_{M+i}$ and $t_{M+1}$, i.e., $\Delta R^{W_{C_{t_{M+i}-t_{M+1}}}}$, is converted from the difference of the rotation vectors of the camera (obtained from the attached tilt sensor) between the time $t_{M+i}$ and $t_{M+1}$, i.e., $\Delta r^{W_{C_{t_{M+i}-t_{M+1}}}}$, by using a Rodrigues formula [58]. The operator ⊕ is denoted as an addition operator between two rotation matrices, where the numerical addition is firstly applied on their corresponding rotation vectors and the Rodrigues conversion is then applied to the result of the numerical addition operations.

However, using the solved camera parameters in Equation (19), the HIVBDM system using a moving camera is still not able to measure the structural displacements $\Delta P_{t_{i}-t_{1}}^{W_{M_{t_{1}}}}$ from $t_{1}$ to $t_{i}$ in the world unit since the camera and structure (moving calibration target) are both subject to translations. Similarly, a stationary structure within the same camera FOV of the structure that is being monitored is used to capture the camera translations since the relative translations between the camera and the stationary structure (stationary calibration target) are considered as pure camera translations.

Given the $L_{t_{i}}$ detected feature points on the stationary calibration target of the input image $I^{t_{i}}$ (green points on the image plane of Figure 3), and their generated spatial locations (blue points in the world coordinate system of Figure 3), the unknown camera parameters, i.e., $A_{S},k_{S},\;R^{W_{S_{t_{i}}}},T^{W_{S_{t_{i}}}}$ in Equation (10), are obtained by minimizing the reprojection error $\left\|\varepsilon_{R}\right\|$ defined in Equation (11). In this study, the estimation of these unknown camera parameters using the stationary calibration target is also considered as a constrained optimization problem, where the camera rotations are known from the attached tilt sensor, and the structure is subject to only translations. Therefore, the constrained optimization problem is defined as follows:

(20) $$\underset{A_{S},k_{S},R^{W_{S_{t_{i}}}},T^{W_{S_{t_{i}}}}}{\min}\|\varepsilon_{R}\|\;s.t.\;R^{W_{S_{t_{M+i}}}}=R^{W_{S_{t_{M+1}}}}⊕\left(\Delta R^{W_{C_{t_{M+i}-t_{M+1}}}}\right),\;\forall i\in \left\{1,\ldots ,N\right\}$$

where the stationary calibration target has the same rotational increments as the moving calibration target in Equation (19).

In Equation (19) and Equation (20), the rotational information obtained from the attached tilt sensor is added as the optimization constraints to the N monitoring images. The constrained optimization problem is iteratively solved by the Levenberg-Marquardt Algorithm [55]. Therefore, based on these solved camera parameters from both the moving and stationary calibration targets, the HIVBDM system using a moving camera is able to measure the structural displacements $\Delta P_{t_{i}-t_{1}}^{W_{M_{t_{1}}}}$ from $t_{1}$ to $t_{i}$ in the world unit.

Similar to those at Section 3.2.1, the measurement process that leverages those obtained camera parameters on both the moving and stationary calibration targets from the N monitoring images is provided in Equations (13)–(18). Eventually, by using a stationary calibration target with an attached tilt sensor as camera movement compensation, the measurement output of the HIVBDM system using a moving camera is shown as follows:

(21) $$\begin{array}{c} \Delta P_{t_{i}-t_{1}}^{W_{M_{t_{1}}}}=R^{W_{M_{t_{1}}}}{}^{-1}R^{W_{S_{t_{1}}}}R^{W_{S_{t_{i}}}}{}^{-1}T^{W_{M_{t_{i}}}}-R^{W_{M_{t_{1}}}}{}^{-1}R^{W_{S_{t_{1}}}}R^{W_{S_{t_{i}}}}{}^{-1}T^{W_{S_{t_{i}}}}\\ +R^{W_{M_{t_{1}}}}{}^{-1}T^{W_{S_{t_{1}}}}-R^{W_{M_{t_{1}}}}{}^{-1}T^{W_{M_{t_{1}}}} \end{array}$$

when the camera is stationary, i.e., $R^{W_{S_{t_{i}}}}\equiv R^{W_{S_{t_{1}}}}$, $T^{W_{S_{t_{i}}}}\equiv T^{W_{S_{t_{1}}}}$, Equation (21) yields the same result as given in Equation (8).

## 4. Experimental Results

**Figure 4:**
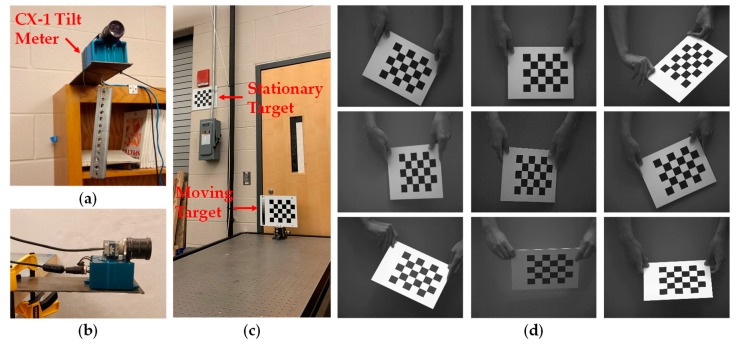
The simulated indoor experimental environment and samples of the captured calibration images in camera calibration: (**a**) utilized moving camera and attached tilt sensor (with weight); (**b**) utilized moving camera and attached tilt sensor (without weight); (**c**) experimental configuration of a stationary and a moving calibration targets; (**d**) samples of the calibration images, where the image intensities need not be constant due to the robust checkerboard corner detections.

In this section, we present the experimental results of the proposed HIVBDM system. The experiments are performed in a laboratory environment, which is shown in Figure 4. This section provides the details and analysis of the components, as follows: (1) the implementation of the camera calibration algorithm is described in Section 4.1; (2) the evaluation of the relative displacement measurements between the camera and target using a stationary camera is presented in Section 4.2; and (3) the evaluation of the relative displacement measurements between the camera and target using a moving camera is presented in Section 4.3.

### 4.1. Implementation of the Camera Calibration Algorithm

The camera calibration algorithm in this study utilizes a planar target with coplanar features, i.e., an empty 30 squares (5 × 6) black and white checkerboard with each square size being equal to 1.25” × 1.25”. Previous studies have suggested using a rigid and flat mounting surface to create a high-quality planar calibration target [54,56]. The planar checkboard calibration targets used, the 2592 × 2048-resolution GigE Genie Nano C2590 camera [59], and the attached CX-1 tilt sensor are shown in Figure 4a.

The input images used in the camera calibration are calibration and monitoring images [54,56]. The calibration images are required in order to obtain a better estimate of the unknown camera parameters described in Equation (1), and those monitoring images are captured as the input for the HIVBDM system for measuring the displacements of the target during the SHM. The general process of acquiring the calibration images includes capturing these images under different target orientations and operating distances. Multiple calibration images that cover the entire camera FOV are encouraged, such that all of the detected feature points within the camera FOV are included in the camera calibration process [54,56]. Samples of these calibration images are shown in Figure 4d. Empirical experience suggests that the entire camera FOV can be covered by either moving the calibration target or moving the camera itself [54]. Andreas Geiger’s algorithm [60] is then applied to detect the corners of the calibration targets, i.e., checkerboards, in those calibration images with sub-pixel accuracy. Please note that the indoor illumination changes shown in Figure 4d do not affect the camera calibration algorithms due to the robust checkboard corner detections [60]. Since the distance between two selected feature points of the checkerboard pattern is known, a ratio R of physical unit to pixel [37] is defined as:

(22) $$R=\frac{d}{D}$$

where d is the pixel distance of square side (33.932 pixels), and D is the length of the square side (31.750 mm). Therefore, the ratio R equals to 1.069 (pixel/mm).

In this study, Root-Mean-Square Error (RMSE) is used as the evaluation metric to evaluate the performance of the relative displacement measurements between the camera and the target [27,39]. The RMSE $\bar{\varepsilon }$ is defined as:

(23) $$\bar{\varepsilon }=\sqrt{\frac{\sum_{i=1}^{N}{\left({\tilde{\Delta }}_{i}-\Delta_{i}\right)}^{2}}{N}}$$

where ${\tilde{\Delta }}_{i}$ is the *i*th measured target displacement, $\Delta_{i}$ is the *i*th ground-truth target displacement and N is the total number of measurements.

### 4.2. Evaluations of the Relative Displacement Measurements between the Camera and Target Using a Stationary Camera

In this section, evaluations of the relative displacement measurements between the camera and target using a stationary camera are reported. A 50 mm lens GigE camera is fixed on the stationary platform in the measurements, and the operating distance between camera and moving calibration target is set to 9.75 m. The displacements in the X and Y directions, i.e., longitudinal and vertical, are considered as “in-plane” translations, and displacements in the Z direction, i.e., towards and away from the camera, are considered to be “out-of-plane” translations. Similarly, ${\bar{\varepsilon }}_{x}$ and ${\bar{\varepsilon }}_{y}$ are termed as “in-plane” RMSE, and ${\bar{\varepsilon }}_{z}$ is termed as “out-of-plane” RMSE. The target is moved to seven different positions in the X, Y and Z directions, respectively. The synthetic target displacements are controlled on an optical table and are measured by a digital caliper with 0.0127 mm (0.0005”) resolution as references. The camera separately captures the static initial position of the target and these seven static target positions. Measuring static target displacements provides the ability to take multiple images of each target position under an assumption that the target and the camera do not move, or the movements are minimal that can be ignored during the image acquisition at each target position. Therefore, to improve the corner detection accuracy, ten different images are taken at each measurement (target position) by the utilized GigE camera with a frame rate of 10 FPS. The detected feature locations of the image shots are averaged before feeding into the camera calibration algorithm. The initial position of the target is set as zero in each of the X, Y and Z directions, and the evaluation results of those synthetic static target displacements using a stationary camera are reported in Table 2.

Although neither the target nor the camera move, or the movements are so minimal that they can be ignored during this image acquisition process, the importance of applying the averaging processing for the feature locations at each target position requires some discussions. Therefore, a comparative analysis for applying the averaging processing for the feature locations is provided in Table 2. The detected feature locations of the first image shot at each target position are fed into the camera calibration algorithm as a comparison.

As shown in Table 2, when comparing the calculated in-plane and out-of-plane RMSE between the cases with and without averaging processing of the detected feature locations at each target position, in-plane RMSE ${\bar{\varepsilon }}_{x}$ and ${\bar{\varepsilon }}_{y}$ displacement measurements in the X direction are obtained with an average of 0.433 mm vs. 0.421 mm, and the out-of-plane RMSE ${\bar{\varepsilon }}_{z}$ is obtained at 1.457 mm vs. 1.604 mm. Moreover, in the Y direction displacement measurements, the in-plane RMSE ${\bar{\varepsilon }}_{x}$ and ${\bar{\varepsilon }}_{y}$ are obtained with an average of 0.142 mm vs. 0.161 mm, and the out-of-plane RMSE ${\bar{\varepsilon }}_{z}$ is obtained at 2.046 mm vs. 2.171 mm. As for the Z direction displacement measurement, the in-plane RMSE ${\bar{\varepsilon }}_{x}$ and ${\bar{\varepsilon }}_{y}$ are obtained at an average of 0.477 mm vs. 0.467 mm, and the out-of-plane RMSE ${\bar{\varepsilon }}_{z}$ is obtained at 0.849 mm vs. 0.625 mm. A comparison of these results indicates that the deviations between these two considered processing variations are trivial, and hence the averaging processing is applied throughout the experiments for consistency.

### 4.3. Evaluations of the Relative Displacement Measurements between the Camera and Target Using a Moving Camera

In this section, a series of experiments is conducted to analyze the performance of relative displacement measurements between the camera and the target using a moving camera as described in Section 3.2. Similar to the measurements given in Section 4.2, a 50 mm camera lens with 9.75 m operating distance between the camera and the moving calibration target was also used for this series of experiments. Also, to capture the camera movements, the distance between the camera and the stationary calibration target was set as 9.85 m. During the displacement measurements, both the stationary and moving calibration targets were required to be placed within the same FOV of the camera.

In Section 4.3.1, the relative displacement measurements between the camera and the target using a moving camera are evaluated with respect to the same seven synthetic static target displacements in each of the X, Y and Z directions, respectively. In Section 4.3.2, an experimental validation of the exact camera movements using a conventional linear variable differential transformer (LVDT) sensor is provided. In Section 4.3.3, the static displacement measurements are evaluated using a long-term indoor monitoring process whereby the moving structure (moving calibration target) is also kept stationary throughout the monitoring process.

#### 4.3.1. Evaluation on the Synthetic Target Displacements

On the synthetic target displacements, the target is moved to seven different positions in the X, Y and Z directions. The synthetic target displacements are controlled on an optical table, and are measured by a digital caliper with 0.0127 mm (0.0005”) resolution as references. As shown in Figure 4a, a GigE camera with an attached CX-1 tilt sensor is fixed above the tip of a cantilever plate, and a weight, i.e., W, is hung underneath the plate to move the camera. The initial position of the target before hanging the weight is set to zero in each of the X, Y and Z direction. The camera captures the static initial position of the target before hanging the weight and those seven static target positions after hanging the weight W. The target displacement measurements are calculated between the initial target position and each of the seven target positions. Meanwhile, the hanging weight rotates the camera support axis and hence rotates and translates the camera. The camera movements mainly come from beam deflection, and can be controlled by using different weights and adjusting different lengths of the cantilever plate. In this study, the hung weight was 0.5 kg, and the length of the cantilever plate to the applied weight was equal to 203 mm. We assume that there is no relative movement between the camera and the attached CX-1 tilt sensor. Therefore, the camera vertical displacement, $\delta_{C}$, is [61]:

(24) $$\delta_{C}=\frac{2}{3}\theta L$$

where θ is the rotation captured by the CX-1 tilt sensor, and L is the length of the cantilever plate to the applied weight. Moreover, to validate the calculated camera movements in Equation (24), a validation of the exact camera movements by using a LVDT sensor is provided in Section 4.3.2.

Measuring the static target displacements follows the assumption that the target and the camera do not move, or that the movements are so minimal that they can be ignored during image acquisition at each target position. As shown in Figure 4c, a stationary calibration target is located near the moving calibration target, such that both the stationary and the moving calibration targets are detected in the same FOV of the camera in each of the captured image. Similar to in Section 4.2, ten different image shots were taken at each target position by the utilized GigE camera with a frame rate of 10 FPS. 

The detected feature locations of the images were averaged before being fed into the camera calibration algorithm. Moreover, during the image capture process, the attached CX-1 tilt-meter records the simultaneous camera rotations. The responses of the camera and the tilt sensor are synchronized based on the timestamps provided by the GigE Camera and the CX-1 tilt sensor. Since the detected feature locations of the image shots at each target position are averaged, the corresponding synchronized camera rotations are averaged accordingly. At each target position, the synchronized-and-averaged camera movements, e.g., rotations and translations, are provided in Table 3 for repeatability. The initial camera position before hanging the weight is set as zero, and the exact camera movements are calculated between the initial camera position and each of the seven camera positions. Please note that based on the limited experimental facilities, only Y direction camera movements are provided as a reference throughout the paper. The evaluation results of those synthetic static target displacements using a moving camera are reported in Table 4.

In Table 4, the camera movement compensation using a stationary calibration target achieves the RMSE at an average of 7.529 mm and 11.832 mm on the in-plane and out-of-plane translations, respectively. By using a supplemental attached tilt sensor, the RMSE is reduced to an average of 1.440 mm and 2.904 mm on the in-plane and out-of-plane translations, respectively. Specifically, using this supplemental attached tilt sensor, the in-plane RMSE ${\bar{\varepsilon }}_{x}$ and ${\bar{\varepsilon }}_{y}$ are decreased from an average of 1.884 mm to 0.852 mm, and from an average of 1.707 mm to 0.702 mm, both on in-plane translations. Similarly, on out-of-plane translations, ${\bar{\varepsilon }}_{x}$ is reduced from 2.107 mm to 1.109 mm, and ${\bar{\varepsilon }}_{y}$ is reduced from 8.846 mm to 3.081 mm by using the supplemental tilt sensor. However, by using only the stationary calibration target in compensating the camera movements, the Z direction measurements of the static target displacements are not accurate, where the out-of-plane RMSE ${\bar{\varepsilon }}_{z}$ is achieved at an average of 18.996 mm on in-plane translations and 24.542 mm on out-of-plane translation. Since the camera rotations captured by the stationary calibration target is less accurate, an attached tilt sensor is used to supplement the stationary calibration target in capturing the camera rotations. Camera movement compensation using a supplemental tilt sensor achieves the least ${\bar{\varepsilon }}_{z}$ on in-plane translations, which is at an average of 2.768 mm, and the ${\bar{\varepsilon }}_{z}$ also achieves the least value (4.522 mm) on out-of-plane translations by using the tilt sensor.

As a result, comparing the measurement results using a moving camera in Table 4 with those using a stationary camera in Table 2, the measurements using a stationary camera show less RMSE than those using a moving camera, in both in-plane and out-of-plane translations. In the measurements using a stationary camera, the in-plane RMSE ${\bar{\varepsilon }}_{x}$ and ${\bar{\varepsilon }}_{y}$ are achieved at an average of 0.350 mm and the out-of-plane RMSE ${\bar{\varepsilon }}_{z}$ is achieved at an average of 1.451 mm, in both in-plane and out-of-plane translations, respectively. Meanwhile, in the measurements using a moving camera where a stationary calibration target with an attached tilt sensor is used as camera movement compensation, the in-plane RMSE ${\bar{\varepsilon }}_{x}$ and ${\bar{\varepsilon }}_{y}$ are increased to an average of 1.216 mm and the out-of-plane RMSE ${\bar{\varepsilon }}_{z}$ is achieved at an average of 3.353 mm, in both in-plane and out-of-plane translations, respectively.

#### 4.3.2. Validation of Exact Camera Movements by Using a LVDT Sensor

In this section, a validation of the exact camera movement measurements given in Equation (24) is provided by using a LVDT sensor (SP2-50 Celesco string potentiometer). The validations are performed on two different weights under three different lengths of cantilever. The validation results are reported in Table 5, where the $\delta_{LVDT}$ is the measurements from the LVDT sensor, the $\delta_{C}$ is the measurements given by Equation (24). The error percentage is calculated between the $\delta_{C}$ and $\delta_{LVDT}$, where $\delta_{LVDT}$ is used as ground truth.

As shown in Table 5, the average of the error percentages across the six test sets between exact camera movements ($\delta_{C}$) and LVDT sensor ($\delta_{LVDT}$) is 5.12% (less than 0.5 mm error in absolute value). Therefore, the validation results show that the exact camera movements given by Equation (24) are close to the camera movements measured by the LVDT sensor.

#### 4.3.3. Evaluation on the Long-Term Indoor Monitoring Process

**Figure 5:**
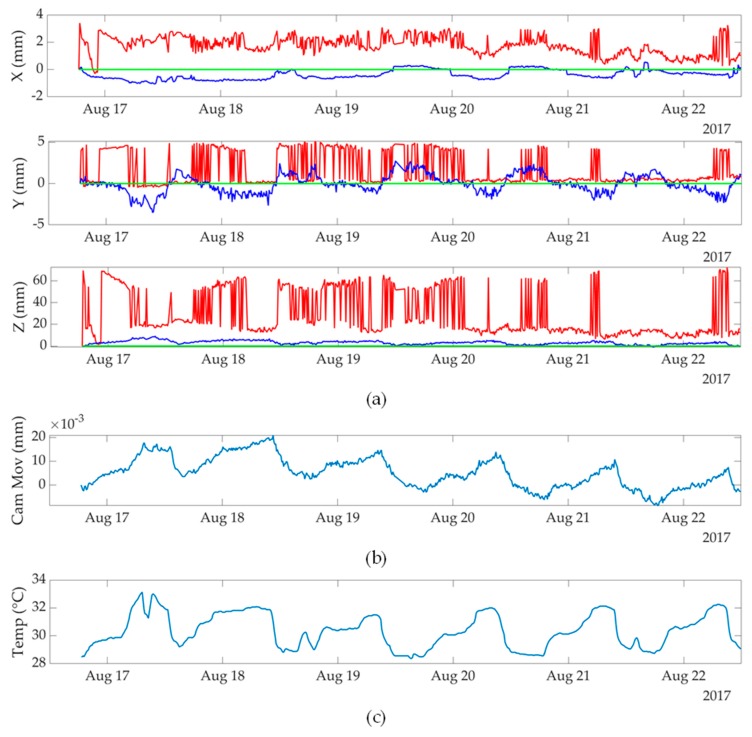
Evaluations of static target displacements in long-term indoor monitoring process using a moving camera: (**a**) static target displacement measurements in the X, Y and Z directions. For the legends, a stationary calibration target is used as the camera movement compensation in the red plots, a stationary calibration target with an attached CX-1 tilt sensor is used as the camera movement compensation in the blue plots, and the green plots show the ground truth target displacements; (**b**) the synchronized and averaged camera movements at each camera capture in the monitoring process; (**c**) the temperatures at each camera capture in the monitoring process.

In the long-term indoor monitoring process, as shown in Figure 4b, without hanging the weight W to move the camera, a 50 mm lens GigE camera with an attached tilt sensor is fixed above a free-moving cantilever plate. The length of cantilever plate to the applied weight also equals to 203 mm. However, without hanging a weight underneath the tip of the cantilever plate, the camera is kept free during the entire monitoring process. In this long-term indoor monitoring process, some environmental effects, such as the temperature changes, causes the length changes of the cantilever, and hence move the camera support. Also, some small activities within the building might also slightly affect the of the camera position on the cantilever. At every ten minutes along the entire monitoring process, i.e., approximately six days, the camera captures the locations of the stationary and moving calibration targets, and the attached CX-1 tilt-meter records the simultaneous camera rotations. Similarly, for each camera capture, the synchronized and averaged camera movements are provided in Figure 5b for repeatability. Meanwhile, in Figure 5c, the temperature history captured by the CX-1 sensor is also provided as a reference. The temperature changes share the similar trends of the camera movements, which indicates that the temperature changes cause length and stiffness changes of the cantilever, and hence moves the camera support and affects the measurements of target displacements. The moving calibration target is kept fixed in this long-term monitoring, and hence the measurement ground truths should indicate that there is zero target displacement in the X, Y, and Z directions of the measurements, respectively.

The numerical results of the static target displacements in the long-term monitoring process are reported in Figure 5a. In the X direction static displacement measurements, the camera movement compensation using a stationary calibration target achieves 1.878 mm RMSE. By using the supplemental attached CX-1 tilt-meter, the RMSE is further decreased to 0.514 mm. Moreover, in the Y direction, static displacement measurements, the camera movement compensation using a stationary calibration target achieves 2.525 mm RMSE. By using the supplemental CX-1 tilt-meter, the RMSE is further decreased to 1.102 mm. In addition, in the Z direction static displacement measurements, the camera movement compensation using a stationary calibration target fails due to the inaccurate camera rotation information. The RMSE of Z direction increases to 35.844 mm by using a stationary calibration target, and an RMSE of 3.578 mm is achieved by using the supplemental tilt sensor.

## 5. Conclusions

This paper presents a novel monocular target-based HIVBDM system that can measure both in-plane and out-of-plane static structural displacements. The proposed HIVBDM system does not require the camera to be stationary during the displacement measurements. Typically, this HIVBDM uses two calibration targets, i.e., one calibration target is kept stationary to compensate camera movements, and the other calibration target is mounted on the surface of the monitored structures in representing the structural displacements. In addition to the stationary calibration target, to further improve the robustness of the HIVBDM system to rotations of the camera, a tilt sensor attached to the camera is used to provide an accurate measurement of the camera rotations. Future research can focus on designing a target-less monocular HIVBDM system that not only supports arbitrary camera movements, but can also accurately measure both the structural translations and rotations. Also, measuring the high-dynamic structural responses will also be considered.

## Figures and Tables

**Table 1:** Frequently used notations in the proposed HIVBDM system.

Symbol	Description
I	Input image sequence from time $t_{1}$ to time $t_{i}$, $I=\left\{I^{t_{1}},\;I^{t_{2}},\ldots ,\;I^{t_{i}}\right\}$
$C_{t_{i}}$	Camera coordinate system at time $t_{i}$
$I_{t_{i}}$	Image plane at time $t_{i}$
$W_{S_{t_{i}}}$	World coordinate system of the stationary structure at time $t_{i}$
$W_{M_{t_{i}}}$	World coordinate system of the moving structure at time $t_{i}$
$W_{C_{t_{i}}}$	World coordinate system of the camera at time $t_{i}$
$A_{S}$	3 × 3 intrinsic camera parameter obtained from the stationary structure
$k_{S}$	1 × 4 camera distortion (warping) parameter obtained from the stationary structure
$A_{M}$	3 × 3 intrinsic camera parameter obtained from the moving structure
$k_{M}$	1 × 4 camera distortion (warping) parameter obtained from the moving structure
$R^{W_{S_{t_{i}}}}$	3 × 3 rotation matrix of the camera in the world coordinate system of the stationary structure at time $t_{i}$
$T^{W_{S_{t_{i}}}}$	3 × 1 translation vector of the camera in the world coordinate system of the stationary structure at time $t_{i}$
$R^{W_{M_{t_{i}}}}$	3 × 3 rotation matrix of the camera in the world coordinate system of the moving structure at time $t_{i}$
$T^{W_{M_{t_{i}}}}$	3 × 1 translation vector of the camera in the world coordinate system of the moving structure at time $t_{i}$
$\Delta r^{W_{C_{t_{j}-t_{i}}}}$	3 × 1 obtained difference of the camera rotation vector from time $t_{i}$ to time $t_{j}$ using an attached tilt sensor
$\Delta R^{W_{C_{t_{j}-t_{i}}}}$	3 × 3 obtained difference of the camera rotation matrix converted from $\Delta r^{W_{C_{t_{j}-t_{i}}}}$ using the Rodrigues formula
${\tilde{p}}_{l}^{I_{S_{t_{i}}}}$	2 × 1 pixel-wise location of the $l^{th}$ detected feature points on the stationary calibration target at time $t_{i}$
${\tilde{p}}_{l}^{I_{M_{t_{i}}}}$	2 × 1 pixel-wise location of the $l^{th}$ detected feature points on the moving calibration target at time $t_{i}$
${\tilde{p}}_{l}^{W_{S_{t_{i}}}}$	3 × 1 spatial location of the $l^{th}$ detected feature points on the stationary calibration target at time $t_{i}$
${\tilde{p}}_{l}^{W_{M_{t_{i}}}}$	3 × 1 spatial location of the $l^{th}$ detected feature points on the moving calibration target at time $t_{i}$
$P_{t_{j}}^{C_{t_{i}}}$	3 × 1 spatial location of the monitored point P at time $t_{j}$ in the camera coordinate system at time $t_{i}$
$P_{t_{j}}^{W_{S_{t_{i}}}}$	3 × 1 spatial location of the monitored point P at time $t_{j}$ in the world coordinate system of the stationary structure at time $t_{i}$
$P_{t_{j}}^{W_{M_{t_{i}}}}$	3 × 1 spatial location of the monitored point P at time $t_{j}$ in the world coordinate system of the moving structure at time $t_{i}$
$\Delta P_{t_{j}-t_{i}}^{W_{M_{t_{k}}}}$	3 × 1 measured structural displacement from time $t_{i}$ to time $t_{j}$ in the world coordinate system of the moving structure at time $t_{k}$
The world coordinate system $W_{M_{t_{i}}}$ is associated with the structure that is being monitored, and the world coordinate system $W_{S_{t_{i}}}$ only exists in the camera movement compensation. The structural displacements can only be calculated within the same coordinate system.	

**Table 2:** Comparative analysis of applying averaging processing to the synthetic static target displacements using a stationary camera (mm).

Actual Static Target Displacements in X, Y and Z Directions			Static Target Displacement Measurements in X, Y and Z Directions					
			With Averaging Processing			Without Averaging Processing		
X	Y	Z	X	Y	Z	X	Y	Z
0.000	0.000	0.000	0.008	−0.029	0.304	0.006	−0.043	0.555
1.588	0.000	0.000	1.719	−0.043	−0.729	1.727	−0.039	−0.797
3.175	0.000	0.000	3.491	−0.131	0.273	3.480	−0.111	−0.138
6.350	0.000	0.000	6.831	−0.133	−0.672	6.829	−0.034	−2.090
12.700	0.000	0.000	13.066	−0.296	0.140	13.075	−0.266	−0.595
25.400	0.000	0.000	26.063	−0.575	1.266	26.061	−0.541	0.740
50.800	0.000	0.000	51.224	−1.039	3.476	51.175	−1.029	3.432
RMSE of X Direction Static Target Measurements:			0.397 (${\bar{\varepsilon }}_{x}$)	0.468 (${\bar{\varepsilon }}_{y}$)	1.457 (${\bar{\varepsilon }}_{z}$)	0.389 (${\bar{\varepsilon }}_{x}$)	0.453 (${\bar{\varepsilon }}_{y}$)	1.604 (${\bar{\varepsilon }}_{z}$)
X	Y	Z	X	Y	Z	X	Y	Z
0.000	0.000	0.000	0.023	0.008	0.295	−0.015	−0.024	0.037
0.000	1.588	0.000	−0.242	1.573	−1.624	−0.220	1.606	−1.430
0.000	3.175	0.000	−0.377	3.281	−2.711	−0.431	3.285	−3.116
0.000	6.350	0.000	−0.142	6.294	−2.115	−0.125	6.287	−2.143
0.000	12.700	0.000	−0.097	12.676	−0.625	−0.276	12.653	−1.973
0.000	25.400	0.000	−0.154	25.527	−1.376	−0.215	25.514	−1.712
0.000	50.800	0.000	−0.246	50.861	−3.533	−0.250	50.871	−3.133
RMSE of Y Direction Static Target Measurements:			0.212 (${\bar{\varepsilon }}_{x}$)	0.071 (${\bar{\varepsilon }}_{y}$)	2.046 (${\bar{\varepsilon }}_{z}$)	0.249 (${\bar{\varepsilon }}_{x}$)	0.073 (${\bar{\varepsilon }}_{y}$)	2.171 (${\bar{\varepsilon }}_{z}$)
X	Y	Z	X	Y	Z	X	Y	Z
0.000	0.000	0.000	0.014	0.039	−0.039	−0.022	0.014	−0.633
0.000	0.000	1.588	−0.030	0.182	1.914	−0.038	0.233	2.606
0.000	0.000	3.175	−0.032	0.194	4.196	−0.050	0.157	3.585
0.000	0.000	6.350	−0.082	0.250	6.144	−0.096	0.217	5.758
0.000	0.000	12.700	−0.104	0.537	13.669	−0.101	0.479	12.856
0.000	0.000	25.400	−0.091	1.012	26.749	−0.105	0.941	25.587
0.000	0.000	50.800	−0.178	1.933	51.845	−0.149	1.935	51.647
RMSE of Z Direction Static Target Measurements:			0.092 (${\bar{\varepsilon }}_{x}$)	0.861 (${\bar{\varepsilon }}_{y}$)	0.849 (${\bar{\varepsilon }}_{z}$)	0.090 (${\bar{\varepsilon }}_{x}$)	0.844 (${\bar{\varepsilon }}_{y}$)	0.625 (${\bar{\varepsilon }}_{z}$)

The negative values represent that the target displacement measurements are as the opposite directions as the actual target displacements.

**Table 3:** The synchronized and averaged static camera movements at each position of the target displacements in the X, Y and Z directions.

Direction of Target Displacements	Test Number	θ	L	$\delta_{C}\;(\mathrm{mm})$
X	1	−0.004	203.200	−0.493
	2	−0.004	203.200	−0.492
	3	−0.004	203.200	−0.493
	4	−0.004	203.200	−0.493
	5	−0.004	203.200	−0.495
	6	−0.004	203.200	−0.497
	7	−0.004	203.200	−0.501
Y	1	−0.004	203.200	−0.498
	2	−0.004	203.200	−0.501
	3	−0.004	203.200	−0.509
	4	−0.004	203.200	−0.500
	5	−0.004	203.200	−0.499
	6	−0.004	203.200	−0.502
	7	−0.004	203.200	−0.504
Z	1	−0.004	203.200	−0.491
	2	−0.004	203.200	−0.501
	3	−0.004	203.200	−0.499
	4	−0.004	203.200	−0.504
	5	−0.004	203.200	−0.497
	6	−0.004	203.200	−0.501
	7	−0.004	203.200	−0.496

Negative $\delta_{C}$ represents that the camera movements are opposite to the Y direction (cantilever beam is concave downward).

**Table 4:** Evaluations on the synthetic static target displacements using a moving camera (mm).

Actual Static Target Displacements in X, Y and Z Directions			Static Target Displacement Measurements in X, Y and Z Directions					
			Using a Stationary Calibration Target			Using a Stationary Calibration Target with an Attached Tilt Sensor		
X	Y	Z	X	Y	Z	X	Y	Z
0.000	0.000	0.000	1.080	−1.699	0.119	−0.479	−0.722	0.961
1.588	0.000	0.000	3.603	−2.122	−1.106	1.567	−1.117	2.857
3.175	0.000	0.000	5.335	−1.836	−5.351	3.071	−0.705	1.565
6.350	0.000	0.000	8.644	−1.567	−7.531	6.223	−0.297	1.860
12.700	0.000	0.000	16.007	−1.801	−9.846	13.529	−0.238	2.762
25.400	0.000	0.000	28.718	−2.634	−8.425	26.260	−1.079	3.055
50.800	0.000	0.000	52.625	−2.233	−10.061	50.478	−0.088	4.479
RMSE of X direction static target measurements:			2.403 (${\bar{\varepsilon }}_{x}$)	2.014 (${\bar{\varepsilon }}_{y}$)	7.129 (${\bar{\varepsilon }}_{z}$)	0.505 (${\bar{\varepsilon }}_{x}$)	0.715 (${\bar{\varepsilon }}_{y}$)	2.726 (${\bar{\varepsilon }}_{z}$)
X	Y	Z	X	Y	Z	X	Y	Z
0.000	0.000	0.000	0.800	−1.376	7.145	−0.650	−0.551	−0.205
0.000	1.588	0.000	0.014	1.159	8.397	−1.203	1.973	−0.906
0.000	3.175	0.000	0.034	3.214	7.902	−0.991	4.133	−1.670
0.000	6.350	0.000	0.592	6.752	8.507	−0.995	7.271	−1.464
0.000	12.700	0.000	−0.270	12.276	7.621	−1.248	13.300	−1.944
0.000	25.400	0.000	0.297	25.588	6.175	−1.069	26.253	−3.710
0.000	50.800	0.000	−3.449	47.445	79.469	−1.872	50.712	−5.650
RMSE of Y direction static target measurements:			1.365 (${\bar{\varepsilon }}_{x}$)	1.399 (${\bar{\varepsilon }}_{y}$)	30.863 (${\bar{\varepsilon }}_{z}$)	1.198 (${\bar{\varepsilon }}_{x}$)	0.688 (${\bar{\varepsilon }}_{y}$)	2.810 (${\bar{\varepsilon }}_{z}$)
X	Y	Z	X	Y	Z	X	Y	Z
0.000	0.000	0.000	1.186	13.767	−32.499	−0.493	−0.561	1.866
0.000	0.000	1.588	3.476	13.146	−36.111	−0.051	0.073	6.874
0.000	0.000	3.175	3.578	13.274	−35.506	−0.016	0.329	8.474
0.000	0.000	6.350	0.013	−1.480	13.960	−0.259	0.744	11.423
0.000	0.000	12.700	0.386	−0.532	21.522	0.429	1.845	18.657
0.000	0.000	25.400	1.671	0.013	32.823	1.523	3.438	29.354
0.000	0.000	50.800	1.359	2.609	57.991	2.406	7.088	53.406
RMSE of Z direction static target measurements:			2.107 (${\bar{\varepsilon }}_{x}$)	8.846 (${\bar{\varepsilon }}_{y}$)	24.542 (${\bar{\varepsilon }}_{z}$)	1.109 (${\bar{\varepsilon }}_{x}$)	3.081 (${\bar{\varepsilon }}_{y}$)	4.522 (${\bar{\varepsilon }}_{z}$)

**Table 5:** Validation results of the exact camera movements by using a LVDT sensor.

Test Number	P	L	θ	$\delta_{LVDT}\;(\mathrm{mm})$	$\delta_{C}\;(\mathrm{mm})$	Error (%)
1	4.900	236.538	0.018	3.048	2.849	6.54%
2	9.800	236.538	0.037	6.350	5.857	7.77%
3	4.900	295.275	0.028	5.588	5.425	2.92%
4	9.800	295.275	0.058	11.938	11.444	4.14%
5	4.900	358.775	0.039	9.906	9.401	5.10%
6	9.800	358.775	0.082	20.574	19.695	4.27%
Please note that the error percentage is defined as $\left|\delta_{C}-\delta_{LVDT}\right|/\delta_{LVDT}$.						
